# Allergy to penicillin and betalactam antibiotics

**DOI:** 10.31744/einstein_journal/2021MD5703

**Published:** 2021-04-15

**Authors:** Mara Morelo Rocha Felix, Marcelo Vivolo Aun, Ullissis Pádua de Menezes, Gladys Reis e Silva de Queiroz, Adriana Teixeira Rodrigues, Ana Carolina D’Onofrio-Silva, Maria Inês Perelló, Inês Cristina Camelo-Nunes, Maria Fernanda Malaman

**Affiliations:** 1 Escola de Medicina e Cirurgia Universidade Federal do Estado do Rio de Janeiro Rio de JaneiroRJ Brazil Escola de Medicina e Cirurgia, Universidade Federal do Estado do Rio de Janeiro, Rio de Janeiro, RJ, Brazil.; 2 Faculdade Israelita de Ciências da Saúde Albert Einstein Hospital Israelita Albert Einstein São PauloSP Brazil Faculdade Israelita de Ciências da Saúde Albert Einstein, Hospital Israelita Albert Einstein, São Paulo, SP, Brazil.; 3 Hospital das Clínicas Faculdade de Medicina de Ribeirão Preto Universidade de São Paulo Ribeirão PretoSP Brazil Hospital das Clínicas, Faculdade de Medicina de Ribeirão Preto, Universidade de São Paulo, Ribeirão Preto, SP, Brazil.; 4 Hospital das Clínicas Universidade Federal de Pernambuco RecifePE Brazil Hospital das Clínicas, Universidade Federal de Pernambuco, Recife, PE, Brazil.; 5 Hospital do Servidor Público Estadual “Francisco Morato de Oliveira” São PauloSP Brazil Hospital do Servidor Público Estadual “Francisco Morato de Oliveira”, São Paulo, SP, Brazil.; 6 Hospital das Clínicas Faculdade de Medicina Universidade de São Paulo São PauloSP Brazil Hospital das Clínicas, Faculdade de Medicina, Universidade de São Paulo, São Paulo, SP, Brazil.; 7 Universidade do Estado do Rio de Janeiro Rio de JaneiroRJ Brazil Universidade do Estado do Rio de Janeiro, Rio de Janeiro, RJ, Brazil.; 8 Escola Paulista de Medicina Universidade Federal de São Paulo São PauloSP Brazil Escola Paulista de Medicina, Universidade Federal de São Paulo, São Paulo, SP, Brazil.; 9 Universidade Tiradentes AracajúSE Brazil Universidade Tiradentes, Aracajú, SE, Brazil.

**Keywords:** Beta-lactams/adverse effects, Penicillins/adverse effects, Anti-bacterial agents/adverse effects, Drug hypersensitivity/diagnosis

## Abstract

Betalactams are the most frequent cause of hypersensitivity reactions to drugs mediated by a specific immune mechanism. Immediate reactions occur within 1 to 6 hours after betalactam administration, and are generally IgE-mediated. They clinically translate into urticaria, angioedema and anaphylaxis. Non-immediate or delayed reactions occur after 1 hour of administration. These are the most common reactions and are usually mediated by T cells. The most frequent type is the maculopapular or morbilliform exanthematous eruption. Most individuals who report allergies to penicillin and betalactams can tolerate this group of antibiotics. To make diagnosis, a detailed medical history is essential to verify whether it was an immediate or non-immediate reaction. Thereafter, *in vivo* and/or *in vitro* tests for investigation may be performed. The challenging test is considered the gold standard method for diagnosis of betalactam hypersensitivity. The first approach when suspecting a reaction to betalactam is to discontinue exposure to the drug, and the only specific treatment is desensitization, which has very precise indications. The misdiagnosis of penicillin allergy affects the health system, since the “penicillin allergy” label is associated with increased bacterial resistance, higher rate of therapeutic failure, prolonged hospitalizations, readmissions, and increased costs. Thus, it is essential to develop strategies to assist the prescription of antibiotics in patients identified with a label of “betalactam allergy” at hospitals, and to enhance education of patients and their caregivers, as well as of non-specialist physicians.

## INTRODUCTION

Betalactam (BL) antibiotics are the first choice of treatment of several infections.^(1)^ They include penicillins, cephalosporins, carbapenems, and monobactams.^(2)^ Among their indications, the role of penicillin stands out in the prevention of rheumatic fever and treatment of syphilis, diseases that are still very prevalent in our country. Penicillin is the drug of choice in the prevention of rheumatic fever, due to its efficacy in the eradication of streptococcus of the oropharynx, low cost, and narrow spectrum of action. In syphilis, penicillin is the drug of choice for all forms, especially neurosyphilis and syphilis in pregnancy, cases in which there are no controlled studies showing the efficacy of other drugs.

Suspected BL allergy leads to the use of alternative antibiotics, which are often less effective, more toxic, more expensive, and may contribute to increased bacterial resistance.^(1)^Thus, the diagnosis of BL allergy is a major public health problem.

The World Health Organization (WHO) defines an adverse drug reaction (ADR) as “any response to a drug which is noxious and unintended, and which occurs at doses normally used in man for prophylaxis, diagnosis, or therapy of disease, or for the modification of physiological function.”^(3)^ Adverse drug reactions are classified as: Type A, which are predictable and related to the direct effects of the drug, and Type B, which are unpredictable, dependent on individual susceptibility, and not directly related to the effects of the drug.^(4,5)^

Drug hypersensitivity reactions (DHR) are defined as reactions clinically similar to allergic reactions, initiated by a defined stimulus, and that can be reproduced.^(3)^ They are subdivided into allergic (mediated by an immune mechanism) and non-allergic (triggered by non-immune mechanisms).^(3)^

Betalactam reactions are the most frequent cause of reactions to drugs mediated by a specific immune mechanism.^(2)^They can be classified as immediate (occur within 1 to 6 hours after administration of the drug) and non-immediate (occur after 1 hour).^(3)^

In the United States, approximately 10% of population refer allergy to penicillin.^(6,7)^ However, after a complete investigation, 90% or more of these patients tolerate penicillin.^(6,7)^ The label of “allergy to penicillin” is associated with prolonged hospitalizations, readmissions, and increased costs.^(8,9)^Thus, all patients with suspected BL hypersensitivity should be evaluated, seeking the correct diagnosis.^(1,2)^On the other hand, diagnostic algorithms have changed in recent years.^(1)^ Thus, the objective of this article was to provide an updated review on BL hypersensitivity. An active search at MEDLINE^®^ and the Latin American and Caribbean Health Sciences Literature (LILACS) databases was performed using the terms “drug hypersensitivity”, “penicillins”, “beta-lactams”, and “diagnosis”, for the period 2000 to 2020. Original articles, reviews, and consensuses on the subject were selected, focusing on the negative impact of the false label of “BL allergy” and the safe use of this group of high efficacy and low cost antibiotics.

## EPIDEMIOLOGY

Epidemiological studies demonstrated the past history of penicillin allergy was reported by about 10% of population who attended healthcare services, and approximately 1.3% reported a history of cephalosporin allergy. These were the two most commonly used classes of BL.^(10,11)^Penicillin was the most often cited medication by patients as causing allergic reactions; it was reported by approximately 12.8% of hospitalized patients.^(12)^

However, when correctly evaluated, only 10% of those who reported penicillin allergy had positive skin tests for the drugs involved, and less than 5% were truly allergic.^(13,14)^ Even so, these reactions are very important, considering that BL hypersensitivity reactions result in longer hospital length of stay and mortality risk.^(15)^

The incidence of IgE-mediated and non IgE-mediated reactions has not increased worldwide over the last 50 years, and a penicillin allergy label has significant consequences for individual and public health.^(14)^ Longitudinal studies from a center in the United States showed the rate of positive penicillin skin tests decreased from 15%, in 1995, to 3%, in 2007, and to 0.8%, in 2013.^(16,17)^

One study in England reported only one case of fatal anaphylaxis by amoxicillin given *per oris*, in 35 years and 100-million treatment courses.^(18)^Another English study showed a prevalence of drug-induced anaphylaxis reported for penicillin of 45.9/10 thousand.^(19)^

Benign skin manifestations, such as urticaria and late maculopapular exanthema, are the most common reactions, but BLs can cause any kind of reaction. The most common cause of acute generalized exanthematous pustulosis (AGEP) are aminopenicillins.^(20)^ In addition, BL may cause severe skin reactions, such as drug reaction with eosinophilia and systemic symptoms (DRESS), Stevens-Johnson syndrome (SJS), and toxic epidermal necrolysis (TEN).^(21)^

In Latin America, a multinational survey showed that BL are the second drug class most associated with hypersensitivity reactions, ranking behind nonsteroidal anti-inflammatory drugs (NSAID).^(22)^ Similarly, when only drug-induced anaphylaxis was investigated in Latin America, BL ranked second, also behind NSAID.^(23)^

## ANTIGENIC DETERMINANTS AND CROSS-REACTIVITY

**Figure 1:**
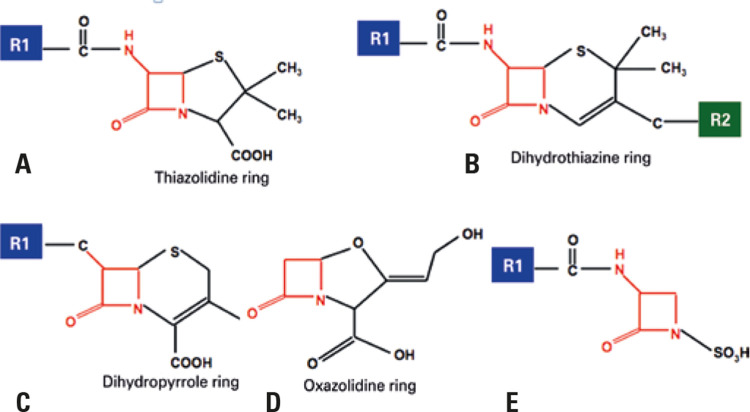
Molecular structure of betalactam antibiotics. (A) Penicillins, (B) Cephalosporins, (C) Carbapenems, (D) Monobactams and (E) Clavulanic acid. Penicillin and the other betalactams share the beta lactam ring (marked in red), but differ from the adjacent ring (absent only in monobactams, shown in image D) and from the lateral chains of group R, R1 (blue), and R2 (green). Betalactams of the penicillin class have only one group R1 (1A). The lateral chain R1 is shared between some penicillins and cephalosporins. Clavulanic acid (E) is a betalactamase inhibitor, formulated with amoxicillin, and has been associated with immunoglobulin E-mediated reactions

Source: Adapted from Castells M, Khan DA, Phillips EJ. Penicillin allergy. N Engl J Med. 2019;381(24):2338-51. Review.^(14)^

The basic chemical structure of BL consists of the following components: BL ring, adjacent ring, and side chains. Penicillins contain one BL ring and an additional ring (thiazolidine), associated with a lateral chain R1. Cephalosporins have a BL ring and an additional ring (dihydrothiazine) associated with two lateral chains, R1 and R2. Carbapenems have one BL ring, one additional ring (dihydropyrrole) and two lateral chains, R1 and R2. Monobactams have only one BL ring associated to a lateral chain R1 (Figure 1).^(24)^

**Figure 2:**
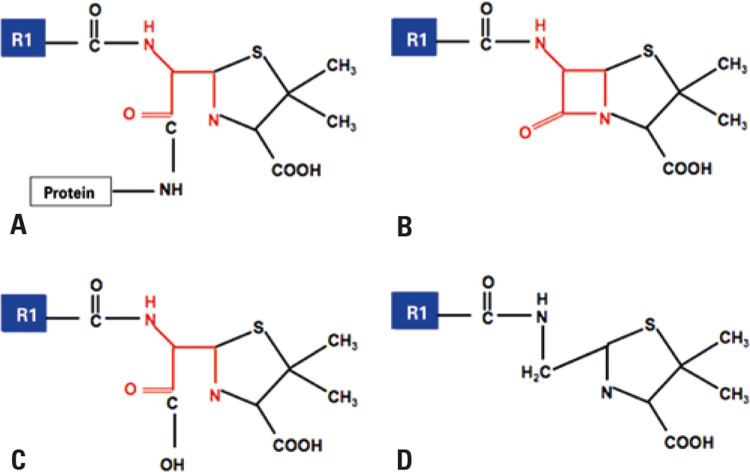
Antigenic determinants of penicillins. Major antigenic determinants (A) Penicilloyl and minor, (B) Penicillin, (C) Penicillinoate and (D) Penilloate

Source: Adapted from Castells M, Khan DA, Phillips EJ. Penicillin allergy. N Engl J Med. 2019;381(24):2338-51. Review.^(14)^

The BL ring, additional rings, and lateral chains are described as potential allergenic sites.^(6)^ Under physiological conditions, the BL ring is unstable and, in the case of penicillins, results in the generation of major and minor determinants, which covalently bind to host proteins (hapten-carrier complex). These determinants are used as test strategies in clinical practice.^(14)^ The BL ring binds to lysine residues in serum proteins and, when it binds to a poly-L-lysine matrix, it creates the major antigenic determinant, penicilloyl- polylysine, which corresponds to 95% of penicillin metabolites. When covalent binding to carboxyl and thiol groups occurs, it generates several minor determinants (5% of metabolites), in which benzylpenicillin, penicillin G, benzylpenicillinoate and benzylpenilloate (Figure 2) are the most important.^(25)^ Unlike penicillins, in which the determinants are stable and identifiable, the allergenic determinants of cephalosporins are not well defined.^(26)^

Cross-reactivity between different BL has been reported in studies, and its approach needs to be made in the context of knowledge of the immune mechanisms involved. IgE class antibodies and T lymphocytes recognize the basic chemical structure associated with carrier proteins. This reactivity among BL depends on the stability of the intermediate products (antigenic determinants) of degradation of BL ring and additional rings.^(14)^Another important factor in cross-reactivity is the structural similarity of lateral chains between BL classes (R1 and R2).^(14)^

The lateral chains of penicillins and first-generation cephalosporins are less complex than the lateral chains of the last-generation cephalosporins; although initial studies indicated more than 5% cross-reactivity between penicillins and cephalosporins, there was suspicion of contamination of cephalosporin preparations with penicillins.^(26)^

Cross-reactivity between penicillins and cephalosporins can be partly predicted with the presence of lateral chain R1 and, to a lesser extent, lateral chains R2. Cross-reactivity between penicillins and cephalosporins seems to be mainly related to the similar or identical lateral chain.^(27)^ Currently, no more than 2% of penicillin-positive patients show reactions to cephalosporins.^(28)^

Regarding other BL, the reactivity between penicillins and carbapenems is less than 1%, and there seems to be no immunologic or clinical cross-reactivity between penicillins and monobactam (aztreonam).^(27,29)^ However, in patients allergic to ceftazidime, there have been reports of reactions to aztreonam, because the lateral chain R1 is identical. Thus, the skin test is recommended in this case.^(27,29)^Similarly, cefazolin, a first-generation cephalosporin widely used in surgical site infection prophylaxis, does not seem to be associated with cross-reactivity with any other BL. In its chemical formula, lateral chains R1 and R2 are not shared with any other BL. This suggests the antigenicity of the antibiotic is defined mainly by the lateral chains, and not by the BL ring or adjacent ring.^(30)^

## IMMUNOLOGICAL MECHANISMS OF REACTIONS TO BETALACTAMS

Betalactam can cause the four types of Gell and Coombs (I, II, III, and IV) hypersensitivity reaction.^(14)^

In the type I reaction (IgE-mediated), dendritic cells bind and internalize the penicillin-bound proteins for presentation of T CD4 + naïve cells. These cells will differentiate into type 2 T cells, with release of interleukin 4 and interleukin 13, inducing the differentiation of B cells and the production of penicillin-specific IgE antibody, which binds to receptors on the surface of basophils and mast cells. In reexposure to penicillin, the activation of these previously sensitized cells induces the degranulation of mast cells and the release of soluble inflammatory mediators, such as tryptase, histamine, prostaglandins, and leukotrienes, leading to immediate clinical manifestations and even anaphylaxis.^(14)^

In type II reactions, the antibody (IgM or IgG) or the immune complex is directed against the structures of the cell membrane of erythrocytes, leukocytes, or platelets, leading to destruction or cell sequestration, including hemolytic anemia and thrombocytopenia.

In type III reactions, antibodies (formed within 4 to 10 days) react with the penicillin transport proteins, generating soluble immune complexes. From then on, there is activation and deposition of the complement in small vessels, leading to recruitment of neutrophils, with release of proteolytic enzymes, generating tissue damage and local vascular inflammation, such as vasculitis in small vessels (hypersensitivity) and serum sickness-like reaction.^(14)^

Late or T-cell-mediated (type IV) reactions occur more than 6 hours after penicillin administration or during treatment, after multiple exposures. An antigen-presenting cell processes drug-modified peptides and presents them at the HLA-antigen binding site for recognition by the T-cell receptor (TCR) in CD4+ or CD8+ T-cells, leading to activation of T-cells and release of cytokines and chemokines.^(31)^

Late reactions may also be related to models involving non-covalent binding, such as the pharmacological interaction model or modification in specificity of the peptide bound to human leukocyte antigens (HLA).^(32)^ These new models are often associated with severe skin reactions with systemic involvement, including SJS, TEN, DRESS, and AGEP.^(14)^ Drug reaction with eosinophilia and systemic symptoms is associated with infiltration of the skin and internal organs by TCD4+, CD8+, and eosinophils. Stevens-Johnson syndrome and TEN depend on CD8+ T cells and are restricted to HLA class I.^(31)^ In AGEP, there is skin infiltration of neutrophils, CD4+, CD8+, and eosinophils.^(14)^

## CLINICAL MANIFESTATIONS

### Immediate reactions

Hypersensitivity reactions to BL can be classified according to the time they occur after drug administration.^(32,33)^ Immediate reactions occur within 1 to 6 hours after BL administration, and are usually mediated by IgE.^(33,34)^They are clinically translated as urticaria, with or without angioedema, and anaphylaxis. Urticaria is characterized by hives (pruritic, transient, erythematous papules disseminated throughout the body).^(33-35)^Angioedema is the edema of the deep dermis, which mainly affects the face (eyelids, lips, ears) and genitalia, and is accompanied by pain and heat.^(35)^ Anaphylaxis is defined as a severe allergic reaction, which can lead to death. The patient may present symptoms, such as pruritus in the palms of hands and soles of feet that become generalized, erythema, urticaria, dyspnea, hypotension, tachycardia, and loss of consciousness.^(33,34)^

### Non-immediate reactions

The non-immediate or late reactions occur after one hour of drug administration and involve a wide spectrum of diseases. They are the most common reactions, generally mediated by T cells.^(3)^The most frequent type is the maculopapular or morbilliform eruption, characterized by erythematous macules and papules, affecting mainly the trunk and proximal extremities. It is observed in about 2% of hospitalized patients, usually appearing 2 to 9 days after initiation of the drug, with a benign course.^(3,36)^

Acute generalized exanthematous pustulosis is a drug reaction that occurs most commonly within 24 to 72 hours after exposure to aminopenicillins. The patient presents with fever, neutrophilic leukocytosis, and generalized exanthema associated with sterile, non-follicular pustules.^(14,35,36)^ There is involvement mainly of the trunk and intertriginous areas. The mortality rate is roughly 4%.^(36)^

Stevens-Johnson syndrome and TEN are severe and painful bullous eruptions occurring 4 to 28 days after administration of the drug.^(14)^ They are characterized by erythematous or purpuric macular exanthema and atypical target lesions, which evolve to blister formation.^(35,36)^ Mucous membranes, most commonly labial, genital, and ocular conjunctive, are involved. The patient presents with fever and is seriously ill. The affected skin extension is <10% in SJS and >30% in TEN, with an overlapping of SJS and TEN, when skin detachment is between 10% and 30%.^(35,36)^ The mortality rate is approximately 10% in SJS, and greater than 30% in TEN.^(36)^

Drug reaction with eosinophilia and systemic symptoms usually occurs 2 to 8 weeks after initiating drug administration, and is associated with fever, macular exanthema, mid-facial edema, lymphadenopathy, eosinophilia, atypical lymphocytosis, and involvement of internal organs (*e.g*., liver, kidneys, lungs and heart).^(14,35,36)^ The mortality rate is approximately 10%. It is interesting to note that DRESS is usually caused by a limited number of drugs, such as anticonvulsants, allopurinol, and sulfonamides.^(37)^ Betalactam are not commonly referred to as cause of this reaction, but there are case reports after the use of this group of antibiotics, mainly cephalosporins.^(37)^ There are studies pointing to the role of viral infections acting as cofactors in this reaction. In particular, there may be replication of the herpes virus group (herpes virus 6 and 7, cytomegalovirus, and Epstein-Barr virus), which have been related to the prolonged course and reactivation of the disease, even after discontinuing the drug.^(35)^

Other late onset hypersensitivity reactions are interstitial nephritis, drug-induced liver injury, cytopenia, and serum sickness-like disease. Serum sickness presents manifestations, such as fever, arthralgias, macular and urticarial exanthema, and lymphadenopathy.^(35)^ Previously, with the use of heterologous serum, its appearance was common 1 to 3 weeks after administration of this immunobiological compound. Currently, penicillins and cephalosporins (especially cefaclor) are the most common causes of serum sickness-like disease, with a latency period of 6 to 8 hours.^(35)^ This clinical presentation is usually self-limited, with an average duration of 1 to 2 weeks.^(36)^

## DIAGNOSIS

### Clinical management

Most individuals who refer allergy to BL can tolerate this group of antibiotics. In any case, a detailed clinical history is fundamental for the evaluation and management of these patients.

The first step is to try to identify if the symptoms are compatible with a possible IgE-mediated mechanism (*e.g*., pruritus, urticaria, angioedema, bronchospasm, laryngeal edema, nausea, vomiting, and hypotension). Additionally, one should ask if the symptoms occurred quickly after the administration of BL (minutes to few hours after the dose). In case of immediate symptoms, probably IgE-mediated, expert evaluation by means of diagnostic tests (skin tests and, if possible, challenge test) is recommended.^(2)^

Another important issue is to verify if symptoms are compatible with a severe non-IgE-mediated mechanism. If the patient has been diagnosed with reactions, such as SJS, TEN, AGEP, DRESS, serum sickness-like disease, immune cytopenia, hepatitis and/or interstitial nephritis, BL exclusion is necessary. Challenge test and/or desensitization are formally contraindicated in these patients.^(2)^

If the history of the reaction is vague or incompatible with an allergic reaction, *e.g*., gastrointestinal intolerance or headache, the skin test is not necessary, and the patient can receive treatment with BL again.^(2)^ On the other hand, if the patient does not know details about the previous reaction, or if was using more than one medication during the episode, a full investigation by means of skin tests and challenge test is recommended.^(2)^

### Skin tests

**Figure 3:**
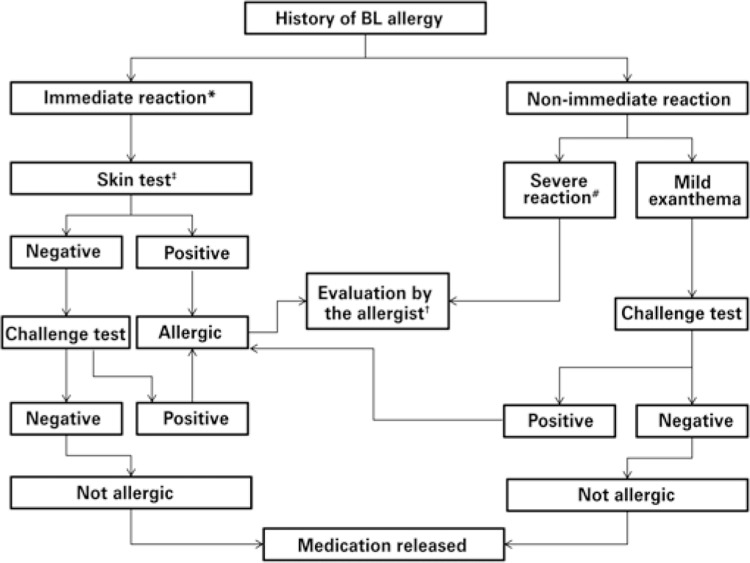
Suggested algorithm for investigation of reactions to betalactams

Allergy to BL should ideally be evaluated when the patient is in good clinical conditions. After taking the history, diagnostic tests can be ordered. In the immediate reactions, the investigation is based on *in vivo* tests (immediate reading skin tests and challenge test) and/or *in vitro* tests (tryptase, specific IgE levels, basophil activation test). In non-immediate reactions, *in vivo* tests include late reading skin tests and challenge test, and the *in vitro* tests comprise lymphocyte transformation test and enzyme-linked immunosorbent spot (ELISPOT). Figure 3 shows the algorithm for evaluation of suspected BL hypersensitivity.

* In severe immediate reactions (anaphylaxis), measure serum specific IgE levels before skin test; ^‡^ skin tests are usually conducted by trained allergy/immunology specialists, in a suitable setting; ^†^ evaluation by allergist can be performed at any time for correct diagnosis, but it is mandatory in cases of already confirmed allergy, in particular for investigation of cross-reactivity between different betalactams, and safe release of class options; ^#^ the severe non-immediate reactions include acute generalized exanthematous pustulosis, drug reaction with eosinophilia, systemic symptoms, Stevens Johnson syndrome, toxic epidermal necrolysis, and the organ-specific reactions (hepatitis, nephritis, hematological, etc.).

BL: betalactams.

Due to the deleterious consequences of the false penicillin allergy label, all patients with a history compatible with an IgE-mediated reaction should be candidates for skin tests.^(2)^In the absence of a clinical history, skin tests should not be used as a screening method.^(2)^Patients with a family history of BL allergy, with no past history of reaction, do not need to be evaluated and can be given BL safely.^(2)^

Immediate reading skin tests (prick, and intradermal) are good methods to evaluate IgE-mediated mechanism in reactions to BL.^(1,32,33,37)^ The prick testing consists of percutaneously crossing the skin with a needle or a puncture device through a solution of the drug. The intradermal test is performed by intracutaneous injection of the drug solution at the concentrations recommended for the test. Both are performed on the volar surface of the forearm. The intradermal test is more sensitive than the prick test, but has greater risk. Thus, the prick test should always precede the intradermal test. They are fast, easy to perform, low cost, and safe. In any case, it is essential that these tests be performed by trained personnel and in a place with adequate support for reversing any anaphylactic reaction. The incidence of adverse systemic reaction during penicillin tests is less than 1%. These tests have no value and should not be performed on patients with a history of severe non IgE-mediated reaction to BL, such as hepatitis, nephritis, SJS, TEN, and severe exfoliative dermatitis.^(38)^Table 1 shows the concentrations recommended for the cutaneous tests (prick and intradermal) with BL.

**Table 1:** Maximum nonirritating concentrations for cutaneous tests (prick and intradermal) with betalactams

Hapten	Prick and intradermal
Benzylpenicillin	10,000IU/mL
Amoxicillin	20mg/mL
Ampicillin	20mg/mL
Cefepime	2mg/mL
Other cephalosporins	20mg/mL
Imipenem	0.5mg/mL
Meropenem	1mg/mL
Aztreonam	2mg/mL

Source: translated and adapted from Romano A, Atanaskovic-Markovic M, Barbaud A, Bircher AJ, Brockow K, Caubet JC, et al. Towards a more precise diagnosis of hypersensitivity to beta-lactams – an EAACI position paper. Allergy. 2020;75(6):1300-15.^(1)^

Late reading skin tests include intradermal and patch skin tests. The intradermal test can be performed with readings of 6 to 8 hours for serum sickness-like disease, or within 48 to 72 hours for type IV reactions.^(39,40)^ The patch test is only useful in type IV reactions. The patch test is considered safer and the main line of investigation of severe reactions (AGEP, SJS, TEN, among others).^(39,40)^ It is performed by means of adhesive strips with small plates containing the suspect drug diluted in petrolatum. Usually, the strips are placed on the patient’s back for 48 hours in the absence of active lesions. In the case of fixed drug eruption, the patch test must be performed on the site where the lesion appeared previously, but not during the acute phase.^(39,40)^

### *In vitro* tests

*In vitro* tests for immediate reactions include tryptase dosing, specific IgE, and basophil activation testing (BAT). Tryptase is dosed in the acute phase of the reaction to determine if there has been mast cell degranulation. Specific IgE measurement and BAT are performed after the acute phase, to identify the drug responsible for the reaction.

Elevated tryptase indicates an anaphylactic type reaction, but does not identify the underlying mechanism.^(41)^ If there is an elevation of tryptase in the acute phase of the reaction, the baseline tryptase must be dosed. An elevation of the baseline tryptase (>11.4μg/L) suggests mastocytosis or a non-clonal mast cell disorder.^(41)^

Specific IgE measurement is commercially available for BL (penicillin G and V, amoxicillin, ampicillin, and some cephalosporins), but has low sensitivity.^(41)^ It is indicated for high-risk patients with immediate hypersensitivity (anaphylaxis, *e.g*.,), before skin tests and challenge test. Basophil activation testing (based on flow cytometry quantification of drug-induced expression of CD63 or CD203c) is available only in specialized centers.^(41)^ It would also play a role in patients with immediate hypersensitivity and high risk, prior to challenge test.

Tests for late (not immediate) reactions include the lymphocytic transformation test, which measures the proliferation of the patient’s T cells over a period of 5 to 7 days, and the ELISPOT, which detects antigen-specific cytokine producing cells after incubation with polymorphonuclear cells for 24 hours.^(42)^ Both tests are performed in the presence of the suspect drugs and only in research centers.

Finally, genetic tests for the evaluation of HLA alleles have been used as screening methods for the prevention of some severe drug reactions. One of the most studied cases is the association between the abacavir-associated hypersensitivity syndrome and the HLA-B*57:01 allele.^(32)^ However, there are no significant genetic associations for immediate allergic reactions to penicillins.^(14)^ Some studies have demonstrated associations between the liver diseases caused by amoxicillin-clavulanate and certain alleles, such as HLA-DRB1*15:01 and HLA-A*02:01, in northern European populations.^(31)^ Nevertheless, due to the low positive predictive value of these alleles for hepatopathies (<1%), these tests have not been used in clinical practice for BL allergy.

### Challenge test/provocation test

Challenge test is defined as the controlled administration of a medication to diagnose reactions to that drug, whether of an immune nature or not. The last American consensus recommends performing the challenge test when there is low probability of hypersensitivity, having as main objective the confirmation of tolerance.^(5)^ The most recent European consensuses consider it the gold standard method in diagnosis of immediate and non-immediate BL hypersensitivity.^(1,33,34)^

Before carrying out the challenge test, it is important to perform risk stratification. Through it, it is possible to define if the patient has high or low risk to react. Table 2 shows the main factors considered in this stratification.^(1)^In any case, some precautions should always be taken: performance in an appropriate place and with a trained team to treat any possible adverse reaction; observation for 1 to 2 hours after the end of the procedure; discontinuation of antihistamines, systemic corticosteroids, or any medication that may interfere with the symptoms during the challenge test (*e.g*., beta-blockers and angiotensin-converting enzyme inhibitors), and an Informed Consent Form signed by the patient.^(43)^

**Table 2:** Risk stratification in betalactam hypersensitivity

Level of risk	Clinical classification of reaction	Clinical picture of reaction
High	Immediate	Anaphylaxis
		Hypotension
		Laryngeal edema
		Bronchospasm
		Urticaria and/or angioedema
		Generalized erythema
	Non-immediate	SJS
		TEN
		DRESS
		AGEP
		Fixed drug eruption, generalized, bullous
		IgA bullous dermatosis
		Severe maculopapular exanthema (confluent rash and progression to erythroderma; duration >1 week; fever, eosinophilia)
		Serum sickness-like disease
		Organ-specific manifestations (cytopenia, nephritis, hepatitis, and pneumonitis)
		Drug-induced autoimmune diseases (lupus, pemphigus vulgaris, and bullous pemphigoid)
Low	Immediate	Isolated generalized pruritus
		Isolated gastrointestinal symptoms (nausea, vomiting, diarrhea)
		Localized urticaria
	Non-immediate	Contact dermatitis
		Local reaction to IM administration
		Palmar exfoliative dermatitis
		Fixed drug eruption
		Late onset urticaria
		Mild to moderate maculopapular exanthema (especially in children)
		SDRIFE

Source: translated and adapted from Romano A, Atanaskovic-Markovic M, Barbaud A, Bircher AJ, Brockow K, Caubet JC, et al. Towards a more precise diagnosis of hypersensitivity to beta-lactams – an EAACI position paper. Allergy. 2020;75(6):1300-15.^(1)^ SJS: Stevens Johnson syndrome; TEN: toxic epidermal necrolysis; DRESS: drug reaction with eosinophilia and systemic symptoms; AGEP: acute generalized exanthematous pustulosis; IM: intramuscular; SDRIFE: symmetrical drug-related intertriginous and flexural exanthema.

Among the relative contraindications for the test are poorly-controlled asthma and active urticaria, whereas the absolute contraindications comprise severe drug reactions and life-threatening anaphylaxis.^(1)^

There are different protocols for the challenge test, but it is often done in two or three steps (placebo, 10% of dose, followed by 90% of dose, after 30 to 60 minutes).^(44)^Amoxicillin is the preferred BL for the test, due to the presence of the BL ring and the R1 lateral chain.^(2)^ In case of suspected amoxicillin-clavulanate allergy, use this medication in the challenge test.^(2)^

Another approach that has been recently evaluated is challenge test with BL, without prior skin tests. Some studies demonstrated the safety of this approach in children with a history of maculopapular exanthema to amoxicillin,^(45)^ and others were performed in adults.^(46)^ However, one of the exclusion criteria used in these studies was history of severe reaction (*e.g.*, anaphylaxis), which contraindicates a direct challenge.

Our group recently published two documents describing the indications, contraindications, and recommended techniques for performing skin tests and drug challenge.^(43,47)^ More information about these tests can be found in the documents. Table 3 summarizes the main *in vivo* and *in vitro* tests for investigation of hypersensitivity to BL.

**Table 3:** *In vivo* and *in vitro* tests for investigation of hypersensitivity to betalactams

Clinical classification of reaction	*In vivo* tests	*In vitro* tests
Immediate reactions	Prick test (immediate reading)	Tryptase
	Intradermal skin test (immediate reading)	Specific IgE
	Challenge test	Basophil activation test
Non-immediate reactions	Patch test	Lymphocyte transformation test
	Intradermal test (late reading)	ELISPOT
	Challenge test	

ELISPOT: enzyme-linked immunosorbent spot.

## TREATMENT

When a reaction to BL is suspected, the first management is to interrupt exposure to the drug.^(3)^ Therapy must be performed according to the clinical syndrome presented by the patient. The only specific treatment for BL hypersensitivity is desensitization, which has very precise indications.

In case of acute urticaria and/or angioedema, antihistamines H1 is the drug of choice, because histamine is the main mediator involved.^(48)^ Preference should be given to second generation antihistamines H1, considering their better safety profile.

In anaphylaxis (severe immediate systemic hypersensitivity reaction that can lead to death), early recognition and rapid treatment are fundamental. Epinephrine is considered the first-line drug for treatment of anaphylaxis, and its early prescription is essential to revert the picture.^(49,50)^

In cases of anaphylaxis, the management is^(49,50)^ to interrupt exposure to the drug; to assess the patient (airways, breathing, circulation, and mental state); to position the patient in supine position, and if possible, to elevate the lower limbs; to call for help of an emergency team; to administer epinephrine 1:1000 (1mg/mL) in doses of 0.01mg/kg (maximum dose of 0.3mg in children and 0.5mg in adults) by intramuscular route, preferably in the vastus lateralis muscle; keep the airway patent, administer supplementary oxygen with a face mask (flow 6 to 8L/minute), and inhaled beta-agonists, if there is bronchospasm; replace intravenous fluids (saline solution 0.9%) and administer second-line drugs, such as antihistamines and corticosteroids.

### Desensitization

When a patient who is allergic to a certain medication needs that same drug for future use and there is no cost-effective substitute, an alternative is desensitization. This is a procedure that allows patients to temporarily tolerate the medication that triggered the original reaction, with administration of the complete dose for treatment.^(5,51,52)^

Desensitization is a precious tool in the management of DHR, especially in the immediate cases, including anaphylaxis, but also in some not severe non-immediate reactions. It can be used in the treatment of any immediate hypersensitivity reaction, allergic or non-allergic. In immediate DHR, rapid drug desensitization (RDD) is used.^(51,53)^

In a short period of time, RDD induces the lack of temporary response to a particular drug that had previously induced a hypersensitivity reaction, allowing the patient to be safely exposed to the culprit drug. This lack of temporary response may be obtained by the gradual reintroduction of small doses of the drug involved up to the total target dose, notably reducing the risk of severe and potentially lethal DHR.^(51)^ Evidence suggests that anaphylaxis effector cells, mast cells, and basophils become transiently hyporresponsive.^(54)^ Clinical tolerance has been described as occurring within a few hours in patients undergoing RDD - a time that does not allow the induction of tolerance at the level of T cells. It has not been established whether repeated RDD in drug-allergic patients could induce regulatory T-cells after multiple desensitizations. The mechanisms that explain the efficacy of desensitization in late reactions are not known. In general, RDD consists of the consecutive administration of small doses of the culprit drug until the total therapeutic dose is reached.

The indications of desensitization to drugs include^(53)^ when there is no alternative drug; the drug involved is more efficient (better quality of life and/or life expectancy) and/or is associated with fewer side effects than alternative drugs, and the culprit drug has a unique mechanism of action, such as acetylsalicylic acid in aspirin-exacerbated respiratory disease.

The procedure is indicated with caution in high-risk patients, and absolutely contraindicated in severe late reactions that are life threatening, such as exfoliative dermatitis, SJS, TEN, DRESS, fixed drug eruption, erythema multiforme, bullous dermatitis, AGEP, severe immunocytotoxic reactions, and vasculitis. This means that after confirming the diagnosis of DHR, the allergist should evaluate the patient’s risk and the risk-benefit ratio of desensitization. When desensitization is indicated, an informed consent should be obtained.^(54)^

The efficacy of desensitization in late reactions is not as well documented as in immediate reactions, and protocols are quite varied, from slow to fast. The first RDD was described with penicillin, during World War II.^(55)^ Since then, the procedure has been used for several classes of drugs, both oral and injectable administration. Among the BL, penicillin is the main example of an antibiotic that can have a precise indication and no clinically effective substitute, such as in the case of syphilis, especially when it affects a pregnant woman.^(53)^ Patients presenting IgE-mediated penicillin allergy, including anaphylaxis, who need penicillin as first-line therapy are candidates for RDD.^(14)^

Oral and parenteral administration routes can be used for RDD, and show similar efficacy, with a success rate of approximately 100%.^(14)^ Some studies suggest that the oral route for patients with penicillin allergy may be safer, easier, and less expensive, although not always the most appropriate. There are protocols combining oral and intravenous RDD for betalactams,^(56)^ and the 12-step intravenous protocol divided into three bags (1:100 dilution, followed by 1:10, and finally the standard dilution) was described as safe and effective, even in patients with severe lung diseases, with forced expiratory volume in 1 second of less than 1L.^(57)^

It is crucial to know that desensitization has a temporary effect that lasts at least two half-lives of the drug, after which the desensitization must be repeated. Long-acting benzathine penicillin is associated with acceptable adverse events one to three weeks after desensitization to penicillin.^(56)^ However, when desensitization is empirical, in the absence of positive skin tests, it does not define whether a patient is truly allergic to the drug. Therefore, it is recommended to perform a formal penicillin allergy test after completing penicillin treatment.^(14)^

## THE IMPORTANCE OF DE-LABELING PENICILLIN ALLERGY

The wrong diagnosis of penicillin allergy can affect the health system in two ways: with the false allergy label, with unreal increase in incidence, and impact on treatment options, and the false non-allergic label, which can have important consequences for the wrong prescription of medications, especially severe reactions.^(58)^

The patient labeled as “penicillin allergy” is more likely to receive broad spectrum antibiotics, such as fluoroquinolones, vancomycin, and clindamycin,^(9,59,60)^ and is at an increased risk for *Clostridium difficile*, methicillin-resistant *Staphylococcus aureus,* and vancomycin-resistant *Enterococcus* infections.^(9,59,60)^ In addition to antimicrobial resistance, studies have shown that patients “allergic to penicillin” have a higher risk of postoperative complications, longer hospital stay, higher cost of treatment, and higher rate of therapeutic failure.^(7,61,62)^Table 4 gathers the main health implications for the individual´s health and public health of the label “BL allergy”.

**Table 4:** Implications for individual´s health and public health of “betalactam allergy” label

Implication for the individual’s health	Implications for the public health system
Fewer options of effective antibiotics	Antimicrobial resistance
More toxic effects associated with the use of alternative antibiotics	High rates of *Clostridium difficile* infection
Use of broad spectrum antibiotics	Use of more expensive antibiotics
More postoperative infections	Increased length of hospital stay

Source: translated and adapted from Castells M, Khan DA, Phillips EJ. Penicillin Allergy. N Engl J Med. 2019;381(24):2338-51. Review.^(14)^

The exaggerated diagnosis of BL allergy stems from overestimated reports, both by physicians and/or other health professionals, and by patients or their guardians. Some symptoms (*e.g*., gastrointestinal intolerance and headache) are falsely considered allergic.^(2)^Additionally, a skin reaction (*e.g*., maculopapular or urticarial exanthema) may be due to the interaction between BL and virus, or even be caused by viral infection.^(2)^ Other considerations that may influence the diagnosis are the “low/suboptimal” sensitivity of skin tests, and the natural history of penicillin allergy.^(2)^ There is a natural decrease of IgE antibodies against penicillin over time.^(2)^

Precise investigation, through appropriate and accurate tests, are essential for the proper management of hypersensitivity reactions to BL.^(57)^ Traditionally, it involves both skin tests and challenge tests. However, direct challenge test without prior skin tests has been increasingly used in low risk patients (mild skin symptoms), aiming to simplify and reduce the cost of diagnostic procedures.^(45,63)^ An American study estimated the cost of investigation through skin tests and challenge tests with penicillin at US$ 220.00. In contrast, direct challenge test would have a cost of US$ 84.00.^(63)^

Some strategies known as “pathways” have been implemented at hospitals around the world to assist in the prescription of antibiotics to patients labeled “BL allergy”.^(1,64)^ These pathways are standardized approaches involving a multidisciplinary team (managers, allergists, infectious disease specialists and general practitioners, nurses, and pharmacists) and aim at rational use of antibiotics.^(1,64)^ Results found generally include lower use of non-BL antibiotics, with lower prevalence of infection by resistant germs; reduction of hospital length of stay; lower mortality rates, and significant reduction of hospital costs.^(1,64)^

Other fundamental aspects are orientation and proper identification of the patient undergoing the full investigation. Many times, even after a negative challenge test, the patient keeps the “allergy” label in the medical records.^(65)^ On some occasions, physicians do not use the medication again, because they do not consider the drug “safe”.^(65)^ Thus, the education of patients and their caregivers, besides non-specialized physicians, is one of the pillars of the “de-labeling” of penicillin allergy.^(66)^

## PARTICULARITIES OF BETALACTAM ALLERGY IN CHILDREN

The study of DHR in children shows large gaps related to epidemiology, clinical aspects, and diagnostic methods. Most children with suspected DHR are not truly allergic to drugs. There are five guidelines for DHR in adults and only one for the pediatric range, and experiences in adults are often extrapolated to children.^(45,67)^

The most common cause of DHR in the pediatric age range is antibiotics, especially BL. In this age group, there are periods in which infections are more frequent (mainly viral infections) and can therefore mimic drug allergies.^(45)^

Accurate assessment of DHR to antibiotics is an essential part of the antibiotic administration program efforts, since removing labels from a patient with a suspected drug allergy can reduce unnecessary exclusions. There are several recent publications with challenge tests using BL without skin tests to guide the decision for re-exposure in individuals, especially children, with mild maculopapular erythema or late onset urticaria.^(68,69)^Finally, in a recent article, the pediatric task force of the European Academy of Allergy and Clinical Immunology (EAACI), together with the British Society for Allergy & Clinical Immunology (BSACI), endorsed the performance of challenge tests in the manifestations mentioned above.^(1)^

In challenge tests, one tenth of the total concentration of BL should be administered initially and, if tolerated, a full dose should be administered for 1 to 7 days later, depending on the time interval of the index reaction (time between the administration of BL and the beginning of the referred reaction). In a challenge test, a non-immediate reaction can be excluded if, after the therapeutic dose has been given, the same time interval as the index reaction has elapsed without the appearance of symptoms. Patients are also advised to return and report if any reaction occurrs in this time interval and preferably show photographs.^(1)^

The penicillin skin test is safe and effective in evaluating children with a history of penicillin allergy. When the diagnosis is of children with a history of benign skin eruptions, but with no report of anaphylaxis, the studies carried out so far examining the direct challenge test to penicillin have been performed by specialists, in emergency departments. The safety of such challenge tests is unknown when performed in non-specialized clinics and adult populations.^(14)^

## CONCLUSION

Betalactams are the most frequent cause of drug hypersensitivity mediated by an immunological mechanism. The label “betalactam allergy” is a major public health problem, because it leads to the use of alternative antibiotics, which are less effective, more toxic, more expensive, and can contribute to increased bacterial resistance. Thus, new strategies have been developed to improve the investigation of hypersensitivity reactions to betalactams. The use of the direct challenge test in low-risk patients tends to increase in the coming years, but it should always be supervised by trained allergists. In addition, the development of educational programs, both for the lay public and for non-specialist healthcare professionals, with the aim to improve recognition, diagnosis, and treatment of hypersensitivity reactions to betalactams is of paramount importance.
