# Supplementary material for: Allergy to penicillin and betalactam antibiotics
Source: Einstein (Sao Paulo). 2021 Apr 15;19:eMD5703. doi: 10.31744/einstein_journal/2021MD5703 (PMC8054529; doi:10.31744/einstein_journal/2021MD5703)
Supplement: Translation

# Translation: Alergia a penicilina e antibióticos beta-lactâmicos

**Authors:** Mara Morelo Rocha Felix, Marcelo Vivolo Aun, Ullissis Pádua de Menezes, Gladys Reis e Silva de Queiroz, Adriana Teixeira Rodrigues, Ana Carolina D’Onofrio-Silva, Maria Inês Perelló, Inês Cristina Camelo-Nunes, Maria Fernanda Malaman

**Affiliations:** 1 Brasil Escola de Medicina e Cirurgia, Universidade Federal do Estado do Rio de Janeiro, Rio de Janeiro, RJ, Brasil.; 2 Brasil Faculdade Israelita de Ciências da Saúde Albert Einstein, Hospital Israelita Albert Einstein, São Paulo, SP, Brasil.; 3 Brasil Hospital das Clínicas, Faculdade de Medicina de Ribeirão Preto, Universidade de São Paulo, Ribeirão Preto, SP, Brasil.; 4 Brasil Hospital das Clínicas, Universidade Federal de Pernambuco, Recife, PE, Brasil.; 5 Brasil Hospital do Servidor Público Estadual “Francisco Morato de Oliveira”, São Paulo, SP, Brasil.; 6 Brasil Hospital das Clínicas, Faculdade de Medicina, Universidade de São Paulo, São Paulo, SP, Brasil.; 7 Brasil Universidade do Estado do Rio de Janeiro, Rio de Janeiro, RJ, Brasil.; 8 Brasil Escola Paulista de Medicina, Universidade Federal de São Paulo, São Paulo, SP, Brasil.; 9 Brasil Universidade Tiradentes, Aracajú, SE, Brasil.

**Keywords:** Beta-lactamas/efeitos adversos, Penicilinas/efeitos adversos, Antibacterianos/efeitos adversos, Hipersensibilidade a drogas/diagnóstico

## Abstract

Os beta-lactâmicos constituem a causa mais frequente de reações de hipersensibilidade a fármacos mediadas por mecanismo imunológico específico. As reações imediatas ocorrem em 1 até 6 horas após a administração do beta-lactâmico, sendo geralmente IgE-mediadas. Elas se traduzem clinicamente por urticária, angioedema e anafilaxia. As reações não imediatas ou tardias ocorrem após 1 hora da administração. São as reações mais comuns, sendo geralmente mediadas por células T. O tipo mais frequente é o exantema maculopapular ou morbiliforme. A maioria dos indivíduos que refere alergia aos beta-lactâmicos pode tolerar esse grupo de antibióticos. No diagnóstico, uma história clínica detalhada é fundamental para verificar se a reação foi do tipo imediato ou não imediato. A partir daí, podem ser realizados testes *in vivo* e/ou *in vitro* para investigação. O teste de provocação é considerado o método padrão-ouro no diagnóstico de hipersensibilidade aos beta-lactâmicos. A primeira conduta diante da suspeita de uma reação ao beta-lactâmico é suspender a exposição ao medicamento, e o único tratamento específico é a dessensibilização, que possui indicações bem precisas. O diagnóstico equivocado de alergia à penicilina afeta o sistema de saúde, pois o rótulo de “alergia à penicilina” está associado a aumento da resistência bacteriana, maior índice de falha terapêutica, hospitalizações prolongadas, readmissões e aumento dos custos. Assim, torna-se fundamental elaborar estratégias com o objetivo de auxiliar na prescrição de antibióticos em pacientes com rótulo de “alergia aos beta-lactâmicos” nos hospitais e melhorar a educação dos pacientes e seus responsáveis, além de médicos não especialistas.

## INTRODUÇÃO

Os antibióticos beta-lactâmicos (BL) são a primeira escolha para tratamento de diversas infecções.^(1)^ Eles incluem as penicilinas, as cefalosporinas, os carbapenêmicos e os monobactâmicos.^(2)^ Entre suas indicações, destaca-se o papel da penicilina na prevenção da febre reumática e no tratamento da sífilis, doenças ainda bastante prevalentes em nosso meio. A penicilina é a droga de escolha na prevenção da febre reumática, devido à sua eficácia na erradicação do estreptococo da orofaringe, ao baixo custo e ao espectro de ação estreito. Na sífilis, a penicilina é a droga de escolha para todas as formas, especialmente a neurossífilis e a sífilis na gestação, casos em que não existem estudos controlados mostrando eficácia de outros fármacos.

A suspeita de alergia aos BL leva ao uso de antibióticos alternativos, muitas vezes menos eficazes, mais tóxicos, mais caros e que podem contribuir para o aumento da resistência bacteriana.^(1)^ Assim, o diagnóstico de alergia ao BL constitui grande problema de saúde pública.

A Organização Mundial da Saúde (OMS) define uma reação adversa a medicamento (RAM) como “qualquer efeito não terapêutico decorrente do uso de um fármaco nas doses habitualmente empregadas para prevenção, diagnóstico ou tratamento de doenças”.^(3)^ As RAM são classificadas como: previsíveis (Tipo A), aquelas relacionadas aos efeitos diretos do fármaco, e imprevisíveis (Tipo B), que são dependentes da suscetibilidade individual e não relacionadas diretamente aos efeitos do fármaco.^(4,5)^

As reações de hipersensibilidade a medicamentos (RHM) são definidas como reações semelhantes clinicamente às reações alérgicas, sendo iniciadas por um estímulo definido e que podem ser reproduzidas.^(3)^ Elas são subdivididas em alérgicas (mediadas por um mecanismo imunológico) e não alérgicas (desencadeadas por mecanismos não imunológicos).^(3)^

As reações aos BL constituem a causa mais frequente de reações a fármacos mediadas por mecanismo imunológico específico.^(2)^ Podem ser classificadas em imediatas (ocorrem em 1 até 6 horas após a administração do medicamento) e não imediatas (acontecem após 1 hora).^(3)^

Nos Estados Unidos, cerca de 10% da população refere alergia à penicilina.^(6,7)^ Entretanto, após investigação completa, 90% ou mais desses pacientes toleram a penicilina.^(6,7)^ O rótulo de “alergia à penicilina” está associado a hospitalizações prolongadas, readmissões e aumento dos custos.^(8,9)^ Assim, todos os pacientes com suspeita de hipersensibilidade a BL devem ser avaliados, buscando-se o diagnóstico correto.^(1,2)^ Por outro lado, os algoritmos diagnósticos sofreram mudanças nos últimos anos.^(1)^ Dessa forma, o objetivo deste artigo foi fornecer uma revisão atualizada sobre a hipersensibilidade aos BL. Foi realizada busca ativa nas bases de dados MEDLINE^®^ e Literatura Latino-Americana e do Caribe em Ciências da Saúde (LILACS), utilizando-se os termos “*drug hypersensitivity*”, “*penicillins*”, “*beta-lactams*” e “*diagnosis*” entre os anos 2000 e 2020. Foram selecionados artigos originais, revisões e consensos sobre o tema, com enfoque no impacto negativo do falso rótulo de “alergia a BL” e no uso seguro desse grupo de antibióticos de elevada eficácia e baixo custo.

## EPIDEMIOLOGIA

Estudos epidemiológicos demonstraram que a história prévia de alergia a penicilinas foi reportada por cerca de 10% da população que frequentou serviços de saúde, e aproximadamente 1,3% referiu história de alergia a cefalosporinas, sendo estas as duas classes de BL mais utilizadas.^(10,11)^ A penicilina foi a medicação mais citada pelos pacientes como causadora de reações alérgicas, sendo relatada por aproximadamente 12,8% dos pacientes hospitalizados.^(12)^

Entretanto, quando avaliados corretamente, apenas 10% daqueles que relataram alergia à penicilina tiveram testes cutâneos positivos para os medicamentos envolvidos, e menos de 5% foram verdadeiramente alérgicos.^(13,14)^ Ainda assim, essas reações possuem grande importância, tendo em vista que as reações de hipersensibilidade aos BL resultam em aumento no tempo de hospitalização e risco de mortalidade.^(15)^

A incidência de reações mediadas ou não por IgE não aumentou em todo o mundo nos últimos 50 anos, e um rótulo de alergia à penicilina tem consequências significativas para a saúde individual e pública.^(14)^ Estudos longitudinais de um centro americano mostraram que a taxa de testes cutâneos positivos de penicilina diminuiu de 15%, em 1995, para 3%, em 2007, e para 0,8%, em 2013.^(16,17)^

Um estudo da Inglaterra relatou apenas um caso de anafilaxia fatal por amoxicilina oral em 35 anos e 100 milhões de cursos de tratamento.^(18)^ Outro estudo inglês demonstrou prevalência de anafilaxia induzida por drogas relatada para penicilina de 45,9/10 mil.^(19)^

As manifestações cutâneas benignas, como urticária e exantema maculopapular tardio, são as reações mais comuns, mas os BL podem causar qualquer tipo de reação. A causa mais comum da pustulose exantematosa generalizada aguda (PEGA) são as aminopenicilinas.^(20)^ Além disso, os BL podem provocar reações cutâneas graves, como reação medicamentosa com eosinofilia e sintomas sistêmicos (DRESS - *drug reaction with eosinophilia and systemic symptoms*), síndrome de Stevens-Johnson (SSJ) e necrólise epidérmica tóxica (NET).^(21)^

Na América Latina, um levantamento multinacional mostrou que os BL são a segunda classe de medicamentos mais associada a reações de hipersensibilidade, perdendo apenas para os anti-inflamatórios não esteroidais (AINE).^(22)^ Da mesma forma, quando se investigou apenas anafilaxia induzida por drogas na América Latina, os BL ocuparam a segunda posição, também atrás dos AINE.^(23)^

## DETERMINANTES ANTIGÊNICOS E REATIVIDADE CRUZADA

A estrutura química básica dos BL consiste na presença dos seguintes componentes: anel BL, anel adjacente e cadeias laterais. As penicilinas contêm um anel BL e um anel adicional (tiazolidina) associado a uma cadeia lateral R1. As cefalosporinas apresentam um anel BL e um anel adicional (dihidrotiazina), associado a duas cadeias laterais R1 e R2. Os carbapenens possuem um anel BL, um anel adicional (*dihidropirrole*) e duas cadeias laterais R1 e R2. Já os monobactans apresentam apenas um anel BL associado à uma cadeia lateral R1 (Figura 1).^(24)^

**Figura 1:** Estrutura molecular dos antibióticos beta-lactâmicos. (A) Penicilinas, (B) Cefalosporinas, (C) Carbapenens, (D) Monobactans e (E) Ácido clavulânico. A penicilina e os demais beta-lactâmicos compartilham o anel beta-lactâmico (assinalado em vermelho), mas diferem em relação ao anel adjacente (ausente apenas nos monobactans, evidenciado na imagem D) e às cadeias laterais do grupo R, R1 (azul) e R2 (verde). Os beta-lactâmicos da classe penicilina possuem apenas um grupo R1 (1A). A cadeia lateral R1 é compartilhada entre algumas penicilinas e cefalosporinas. O ácido clavulânico (E) é um beta-lactâmico que também é um inibidor da beta-lactamase, é formulado com amoxicilina e foi associado a reações mediadas pela imunoglobulina E

Fonte: adaptado de Castells M, Khan DA, Phillips EJ. Penicillin allergy. N Engl J Med. 2019;381(24):2338-51. Review.^(14)^

O anel BL, os anéis adicionais e as cadeias laterais são descritos como potenciais sítios alergênicos.^(6)^ Sob condições fisiológicas, o anel BL é instável e, no caso das penicilinas, resulta na geração de determinantes principais e secundários, que se ligam covalentemente a proteínas do hospedeiro (complexo hapteno-carreador). Esses determinantes são usados como estratégias de teste na prática clínica.^(14)^ O anel BL se liga aos resíduos de lisina em proteínas séricas e, quando se liga a uma matriz de polilisina, cria o determinante antigênico principal, a peniciloil polilisina, que corresponde a 95% dos metabólitos da penicilina. Quando ocorre a ligação covalente aos grupos carboxil e tiol, gera vários determinantes secundários (5% dos metabólitos), sendo a benzilpenicilina, (penicilina G), o benzilpeniciloato e o benzilpeniloato (Figura 2) os mais importantes.^(25)^ Ao contrário das penicilinas, nas quais os determinantes são estáveis e identificáveis, os determinantes alergênicos das cefalosporinas não estão bem definidos.^(26)^

**Figura 2:** Determinantes antigênicos das penicilinas. Determinantes antigênicos principal (A) Peniciloil e secundários, (B) Penicilina, (C) Peniciolato e (D) Peniloato

Fonte: adaptado de Castells M, Khan DA, Phillips EJ. Penicillin allergy. N Engl J Med. 2019;381(24):2338-51. Review.^(14)^

A reatividade cruzada entre os diferentes BL tem sido relatada em estudos, e sua abordagem precisa ser feita no contexto do conhecimento dos mecanismos imunológicos envolvidos. Anticorpos da classe IgE e linfócitos T reconhecem a estrutura química básica associada a proteínas carreadoras. Essa reatividade entre os BL depende da estabilidade dos produtos intermediários (determinantes antigênicos) da degradação dos anéis BL e anéis adicionais.^(14)^ Outro fator importante na reatividade cruzada é a semelhança estrutural das cadeias laterais entre as classes dos BL (R1 e R2).^(14)^

As cadeias laterais das penicilinas e cefalosporinas de primeira geração são menos complexas do que as cadeias laterais das cefalosporinas de última geração e, embora estudos iniciais indicassem mais de 5% de reatividade cruzada entre penicilinas e cefalosporinas, havia suspeita de contaminação das preparações de cefalosporinas com penicilinas.^(26)^

A reatividade cruzada entre penicilinas e cefalosporinas pode, em parte, ser prevista com a presença de cadeia lateral R1 e, em menor grau, das cadeias laterais R2. A reatividade cruzada entre as penicilinas e as cefalosporinas parece estar principalmente relacionada à cadeia lateral semelhante ou idêntica.^(27)^ Atualmente, não mais de 2% dos pacientes com teste positivo para as penicilinas apresentam reação às cefalosporinas.^(28)^

Em relação a outros BL, a reatividade entre penicilinas e carbapenens é menor que 1% e parece não haver reatividade cruzada imunológica ou clínica entre penicilinas e monobactam (aztreonam).^(27,29)^ No entanto, em pacientes alérgicos à ceftazidima, houve relatos de reações ao aztreonam, devido à cadeia lateral R1 ser idêntica. Dessa forma, o teste cutâneo é recomendado nesse caso.^(27,29)^ Da mesma forma, a cefazolina, cefalosporina de primeira geração, muito utilizada em profilaxia de infecção de sítio cirúrgico, parece não estar associada à reatividade cruzada com nenhum outro BL. Em sua fórmula química, as cadeias laterais R1 e R2 não são compartilhadas com nenhum BL. Isso sugere que a antigenicidade do antibiótico é definida principalmente pelas cadeias laterais, e não pelo anel BL ou anel adjacente.^(30)^

## MECANISMOS IMUNOLÓGICOS DAS REAÇÕES AOS BETA-LACTÂMICOS

Os BL podem causar os quatro tipos de reação de hipersensibilidade de Gell e Coombs (I, II, III e IV).^(14)^

Na reação do tipo I (mediada por IgE), as células dendríticas se ligam e internalizam as proteínas ligadas à penicilina para apresentação das células T CD4 + *naive*. Essas células se diferenciarão em células T tipo 2, com liberação de interleucina 4 e interleucina 13, induzindo à diferenciação de células B e à produção de anticorpo IgE específico para penicilina, que se liga a receptores na superfície dos basófilos e mastócitos. Na reexposição à penicilina, a ativação dessas células previamente sensibilizadas induz à desgranulação de mastócitos e à liberação de mediadores inflamatórios solúveis, como triptase, histamina, prostaglandinas e leucotrienos, levando a manifestações clínicas imediatas e podendo configurar até anafilaxia.^(14)^

Nas reações do tipo II, o anticorpo (IgM ou IgG) ou o complexo imunológico se direciona contra as estruturas da membrana celular de eritrócitos, leucócitos ou plaquetas, levando à destruição ou ao sequestro celular, incluindo anemia hemolítica e trombocitopenia.

Nas reações do tipo III, os anticorpos (formados dentro de 4 a 10 dias) reagem com as proteínas transportadoras da penicilina, gerando complexos imunes solúveis. A partir daí, há ativação e deposição de complemento em pequenos vasos, que levam ao recrutamento de neutrófilos, com liberação de enzimas proteolíticas, gerando dano tecidual e inflamação vascular local, como vasculite em pequenos vasos (hipersensibilidade) e doença do soro-símile.^(14)^

As reações tardias ou mediadas por células T (tipo IV) ocorrem mais de 6 horas após a administração de penicilina ou durante o curso de tratamento após múltiplas exposições. Uma célula apresentadora de antígeno processa peptídeos modificados por drogas e os apresenta no sítio de ligação ao antígeno do HLA para reconhecimento pelo receptor de células T (TCR) nas células T CD4+ ou CD8+, levando à ativação de células T e à liberação de citocinas e quimiocinas.^(31)^

As reações tardias também podem estar relacionadas com modelos que envolvem ligação não covalente, como o modelo de interação farmacológica ou alteração da especificidade do peptídeo ligado aos antígenos leucocitários humanos (HLA).^(32)^ Esses novos modelos frequentemente associam-se a reações cutâneas graves com envolvimento sistêmico, incluindo SSJ, NET, DRESS e PEGA.^(14)^ A DRESS está associada à infiltração da pele e órgãos internos por células TCD4+, CD8+ e eosinófilos. A SSJ e a NET são dependentes de células T CD8+ e restritas a HLA classe I.^(31)^ Na PEGA, ocorre infiltração cutânea de neutrófilos, células T CD4+, T CD8+ e eosinófilos.^(14)^

## MANIFESTAÇÕES CLÍNICAS

### Reações imediatas

As reações de hipersensibilidade aos BL podem ser classificadas de acordo com o tempo em que ocorrem após a administração da droga.^(32,33)^ As reações imediatas ocorrem em até 1 a 6 horas após a administração do BL, sendo geralmente IgE-mediadas.^(33,34)^ Elas se traduzem clinicamente por urticária, com ou sem angioedema, e anafilaxia. A urticária caracteriza-se por urticas (pápulas eritematosas pruriginosas, transitórias, disseminadas pelo corpo).^(33-35)^ O angioedema é o edema da derme profunda, que atinge principalmente face (pálpebras, lábios, orelhas) e genitália, sendo acompanhado de dor e calor.^(35)^ A anafilaxia é definida como uma reação alérgica grave, de início rápido e que pode levar ao óbito. O paciente pode apresentar sintomas como prurido nas palmas e plantas que se torna generalizado, eritema, urticária, dispneia, hipotensão, taquicardia e perda da consciência.^(33,34)^

### Reações não imediatas

As reações não imediatas ou tardias ocorrem após uma hora da administração da droga e envolvem um amplo espectro de doenças. São as reações mais comuns, sendo geralmente mediadas por células T.^(3)^ O tipo mais frequente é a erupção maculopapular ou morbiliforme, caracterizada por máculas e pápulas eritematosas, afetando principalmente tronco e extremidades proximais. É observada em cerca de 2% dos pacientes hospitalizados, geralmente aparece após 2 a 9 dias da introdução da droga e tem curso benigno.^(3,36)^

A PEGA é uma farmacodermia que ocorre mais comumente dentro de 24 a 72 horas após a exposição a aminopenicilinas. O paciente apresenta febre, leucocitose neutrofílica e exantema generalizado associado a pústulas estéreis, não foliculares.^(14,35,36)^ Há envolvimento principalmente do tronco e áreas intertriginosas. A taxa de letalidade está em torno de 4%.^(36)^

A SSJ e a NET são erupções bolhosas graves e dolorosas que ocorrem 4 a 28 dias após a administração do medicamento.^(14)^ São caracterizadas por exantema macular eritematoso ou purpúrico e lesões em alvo atípicas, que evoluem para formação de bolhas.^(35,36)^ As mucosas, mais comumente labial, genital e na conjuntiva ocular, estão envolvidas. O paciente apresenta febre e encontra-se gravemente doente. A extensão da pele afetada é <10% na SSJ e >30% na NET, com uma sobreposição SSJ e a NET quando o destacamento cutâneo está entre 10% e 30%.^(35,36)^ A taxa de letalidade é cerca de 10% na SSJ e maior que 30% na NET.^(36)^

A DRESS geralmente ocorre de 2 a 8 semanas após o início da administração do medicamento e está associada a febre, exantema macular, edema centrofacial, linfadenopatia, eosinofilia, linfocitose atípica e acometimento de órgãos internos (por exemplo, fígado, rins, pulmões e coração).^(14,35,36)^ A taxa de letalidade é de aproximadamente 10%. É interessante notar que a DRESS geralmente é causada por um número limitado de drogas, como os anticonvulsivantes, alopurinol e sulfonamidas.^(37)^ Os BL não são comumente referidos como causa dessa reação, porém já existem relatos de casos após o uso desse grupo de antibióticos, principalmente as cefalosporinas.^(37)^ Há estudos que apontam para o papel de infecções virais atuando como cofatores nessa reação. Em especial, pode haver replicação dos vírus do grupo herpes (herpes vírus 6 e 7, citomegalovírus e vírus Epstein-Barr), que têm sido relacionados ao curso prolongado e a reativações da doença, mesmo após a suspensão do fármaco.^(35)^

Outras reações de hipersensibilidade de aparecimento tardio são a nefrite intersticial, a lesão hepática induzida por drogas, as citopenias e a doença do soro-símile. A doença do soro apresenta manifestações como febre, artralgias, exantema macular e urticariforme e linfadenopatia.^(35)^ Anteriormente, com o uso de soro heterólogo, era comum seu aparecimento 1 a 3 semanas após a administração desse composto imunobiológico. Atualmente, as penicilinas e as cefalosporinas (em especial, o cefaclor) são as causas mais comuns de doença do soro-símile, com período de latência de 6 a 8 horas.^(35)^ Essa apresentação clínica geralmente é autolimitada, com duração média de 1 a 2 semanas.^(36)^

## DIAGNÓSTICO

### Manejo clínico

A maioria dos indivíduos que refere alergia aos BL pode tolerar esse grupo de antibióticos. De toda forma, uma história clínica detalhada é fundamental para a avaliação e o manejo desses pacientes.

O primeiro passo é tentar identificar se os sintomas são compatíveis com um possível mecanismo IgE-mediado (por exemplo: prurido, urticária, angioedema, broncoespasmo, edema laríngeo, náuseas, vômitos e hipotensão). Além disso, deve-se perguntar se os sintomas ocorreram rapidamente após a administração do BL (minutos a poucas horas após a dose). No caso de sintomas imediatos, provavelmente IgE-mediados, recomenda-se avaliação por especialista por meio de testes diagnósticos (testes cutâneos e, se possível, teste de provocação – TP).^(2)^

Outra questão importante é verificar se os sintomas são compatíveis com um mecanismo não IgE-mediado grave. Caso o paciente tenha tido o diagnóstico de reações como SSJ, NET, PEGA, DRESS, doença do soro-símile, citopenias imunes, hepatite e/ou nefrite intersticial, a exclusão do BL é necessária. A provocação e/ou dessensibilização estão formalmente contraindicadas nesses pacientes.^(2)^

Caso a história da reação seja vaga ou incompatível com uma reação alérgica, por exemplo, intolerância gastrintestinal ou cefaleia, o teste cutâneo não é necessário, e o paciente pode receber tratamento com BL novamente.^(2)^ Por outro lado, se o paciente não souber detalhes sobre sua reação prévia ou se estava usando mais de uma medicação durante o episódio, é mais indicada uma investigação completa por meio de testes cutâneos e TP.^(2)^

### Testes cutâneos

A alergia aos BL deve ser idealmente avaliada quando o paciente estiver em boas condições clínicas. Após a anamnese, podem ser realizados testes diagnósticos. Nas reações imediatas, a investigação baseia-se em testes *in vivo* (testes cutâneos de leitura imediata e TP) e/ou *in vitro* (triptase, dosagem de IgE específica, teste de ativação basofílica). Nas reações não imediatas, os testes *in vivo* incluem os testes cutâneos de leitura tardia e TP, e os testes *in vitro* incluem o teste de transformação linfocítica e o ELISPOT (*enzyme-linked immunosorbent spot*). A figura 3 mostra o algoritmo para avaliação de suspeita de hipersensibilidade ao BL.

**Figura 3:** Algoritmo sugerido para investigação de reações aos beta-lactâmicos

* Nas reações imediatas graves (anafilaxia), fazer dosagem sérica de IgE específica antes do teste cutâneo; ^‡^ os testes cutâneos são geralmente realizados por especialistas em alergia/imunologia, treinados, em ambiente adequado; ^†^ a avaliação de especialista em alergia pode ser realizada em qualquer momento para correto diagnóstico, mas é mandatória nos casos de alergia já confirmada, em particular para investigação de reatividade cruzada entre diferentes beta-lactâmicos e liberação de opção da classe em segurança; ^#^ as reações não imediatas graves incluem pustulose exantemática generalizada aguda, reação medicamentosa com eosinofilia e sintomas sistêmicos, Síndrome de Stevens Johnson, necrólise epidérmica tóxica e as órgão-específicas (hepatite, nefrite, hematológicas etc).

BL: beta-lactâmicos; TP: teste de provocação.

Devido às consequências deletérias do falso rótulo de alergia à penicilina, todos os pacientes com história compatível com uma reação IgE-mediada devem ser candidatos aos testes cutâneos.^(2)^ Na ausência de história clínica, o teste cutâneo não deve ser usado como método de triagem.^(2)^ Os pacientes que possuem história familiar de alergia aos BL, sem história pessoal de reação prévia, não precisam ser avaliados e podem receber BL de forma segura.^(2)^

Os testes cutâneos de leitura imediata (puntura e intradérmico) são bons métodos para avaliação de mecanismo IgE-mediado nas reações aos BL.^(1,32,33,37)^ O teste de puntura consiste em atravessar a pele percutaneamente com uma agulha ou um puntor através de uma solução do medicamento. O teste intradérmico é realizado por meio da injeção intradérmica da solução do medicamento nas concentrações recomendadas para o teste. Ambos são realizados na superfície volar do antebraço. O teste intradérmico é mais sensível que o de puntura, porém tem maior risco, assim a puntura sempre deve preceder o teste intradérmico. São rápidos, de fácil execução, baixo custo e seguros. De qualquer modo, é essencial que esses testes sejam realizados por pessoal treinado e em ambiente com suporte adequado para reversão de eventual reação anafilática. A incidência de reação sistêmica adversa durante testes com penicilina é menor que 1%. Esses testes não têm valor e não devem ser realizados em pacientes com história de reação não IgE-mediada grave aos BL, como, por exemplo, hepatite, nefrite, SSJ, NET e dermatite esfoliativa grave.^(38)^ A tabela 1 mostra as concentrações recomendadas para realização dos testes cutâneos (puntura e intradérmico) com BL.

**Tabela 1:** Concentrações máximas não irritativas para testes cutâneos (puntura e intradérmico) com beta-lactâmicos

Hapteno	Puntura e intradérmico
Benzilpenicilina	10.000UI/mL
Amoxicilina	20mg/mL
Ampicilina	20mg/mL
Cefepime	2mg/mL
Outras cefalosporinas	20mg/mL
Imipenem	0,5mg/mL
Meropenem	1mg/mL
Aztreonam	2mg/mL

Fonte: traduzido e adaptado Romano A, Atanaskovic-Markovic M, Barbaud A, Bircher AJ, Brockow K, Caubet JC, et al. Towards a more precise diagnosis of hypersensitivity to beta-lactams – an EAACI position paper. Allergy. 2020;75(6):1300-15.^(1)^

Os testes cutâneos de leitura tardia incluem o intradérmico e o de contato. O intradérmico pode ser realizado com leituras de 6 a 8 horas, para doença do soro-símile, ou em 48 a 72 horas, para reações do tipo IV.^(39,40)^ Já o teste de contato só é útil nas reações do tipo IV. O teste de contato é considerado mais seguro e constitui a principal linha de investigação das reações graves (PEGA, SSJ, NET, entre outras).^(39,40)^ É realizado por meio de tiras adesivas com pequenas placas contendo o medicamento suspeito diluído em vaselina. Geralmente, é colocado no dorso do paciente por 48 horas na ausência de lesões ativas. No caso de erupção fixa a fármacos, o teste de contato deve ser realizado sobre o local onde a lesão surgiu anteriormente, mas não na fase aguda.^(39,40)^

### Testes *in vitro*

Os testes *in vitro* para reações imediatas incluem a dosagem de triptase, o IgE específica e o teste de ativação de basófilos (TAB). A triptase é dosada na fase aguda da reação para determinar se houve desgranulação mastocitária. A dosagem de IgE específica e o TAB são realizados após a fase aguda, para identificar o fármaco responsável pela reação.

A triptase elevada indica que houve reação do tipo anafilática, porém não identifica o mecanismo subjacente.^(41)^ Caso haja elevação da triptase na fase aguda da reação, deve-se dosar a triptase basal. Uma elevação da triptase basal (>11,4μg/L) sugere mastocitose ou uma desordem não clonal de mastócitos.^(41)^

A dosagem de IgE específica está disponível comercialmente para BL (penicilina G e V, amoxicilina, ampicilina e algumas cefalosporinas), mas possui baixa sensibilidade.^(41)^ É indicada para pacientes de alto risco com hipersensibilidade imediata (anafilaxia, por exemplo), antes dos testes cutâneos e do TP. O TAB (baseado na quantificação por citometria de fluxo da expressão de CD63 ou CD203c induzida por drogas) está disponível apenas em centros especializados.^(41)^ Também teria papel em pacientes com hipersensibilidade imediata e de alto risco, antes do TP.

Os testes para reações tardias (não imediatas) incluem o teste de transformação linfocítica, que mede a proliferação das células T do paciente em um período de 5 a 7 dias, e o ELISPOT, que detecta as células produtoras de citocinas, antígeno-específicas, após a incubação com células polimorfonucleares por 24 horas.^(42)^ Ambos os testes são realizados na presença dos fármacos suspeitos e somente em centros de pesquisa.

Por último, os testes genéticos para avaliação de alelos HLA têm sido utilizados como métodos de triagem para prevenção de certas farmacodermias graves. Um dos casos mais estudados é a associação entre a síndrome de hipersensibilidade pelo antirretroviral abacavir e o alelo HLA-B*57:01.^(32)^ Entretanto, não existem associações genéticas significativas para as reações alérgicas imediatas às penicilinas.^(14)^ Alguns estudos demonstraram associações entre hepatopatias causadas pela amoxicilina-clavulanato e certos alelos, como o HLA-DRB1*15:01 e o HLA-A*02:01 em populações do norte da Europa.^(31)^ Porém, devido ao baixo valor preditivo positivo desses alelos para hepatopatias (<1%), esses testes não têm sido utilizados na prática clínica para alergia aos BL.

### Teste de provocação

O TP é definido como a administração controlada de uma medicação para diagnosticar reações àquela medicação, seja de natureza imunológica ou não. O último consenso americano recomenda a realização do TP quando existe baixa probabilidade de hipersensibilidade, tendo como objetivo principal a confirmação da tolerância.^(5)^ Já os consensos europeus mais recentes consideram-no como o método padrão-ouro no diagnóstico de hipersensibilidade imediata e não imediata aos BL.^(1,33,34)^

Antes da realização do TP, é importante fazer uma estratificação do risco. Por meio dela, é possível definir se o paciente possui alto ou baixo risco para reagir. Na tabela 2, estão os principais fatores considerados nessa estratificação.^(1)^ De todo modo, alguns cuidados devem ser sempre tomados: realização em local apropriado e com equipe treinada para tratamento de eventual reação adversa; observação por 1 a 2 horas após o término do procedimento; suspensão de anti-histamínicos, corticosteroides sistêmicos ou qualquer medicação que possa interferir nos sintomas durante o TP (por exemplo: betabloqueadores e inibidores da enzima conversora da angiotensina) e assinatura de um Termo de Consentimento Livre e Esclarecido pelo paciente.^(43)^

**Tabela 2:** Estratificação de risco na hipersensibilidade aos beta-lactâmicos

Nível de risco	Classificação clínica da reação	Quadro clínico da reação
Alto	Imediatas	Anafilaxia
		Hipotensão
		Edema laríngeo
		Broncoespsamo
		Urticária e/ou angioedema
		Eritema generalizado
	Não imediatas	SSJ
		NET
		DRESS
		PEGA
		Erupção fixa por droga generalizada bolhosa
		Dermatose bolhosa por IgA
		Exantema maculopapular grave (*rash* confluente e evolução para eritrodermia; duração >1 semana; febre, eosinofilia)
		Doença do soro-símile
		Manifestações órgão-específicas (citopenias, nefrite, hepatite e pneumonite)
		Doenças autoimunes induzidas por droga (lúpus, pênfigo vulgar e penfigoide bolhoso)
Baixo	Imediatas	Prurido generalizado isolado
		Sintomas gastrintestinais isolados (náuseas, vômitos, diarreia)
		Urticária localizada
	Não imediatas	Dermatite de contato
		Reação local à administração IM
		Exantema esfoliativo palmar
		Erupção fixa por droga
		Urticária de aparecimento tardio
		Exantema maculopapular leve a moderado (especialmente em crianças)
		SDRIFE

Fonte: traduzido e adaptado: Romano A, Atanaskovic-Markovic M, Barbaud A, Bircher AJ, Brockow K, Caubet JC, et al. Towards a more precise diagnosis of hypersensitivity to beta-lactams – an EAACI position paper. Allergy. 2020;75(6):1300-15.^(1)^ SSJ: síndrome de Stevens Johnson; NET: necrólise epidérmica tóxica; DRESS: reação medicamentosa com eosinofilia e sintomas sistêmicos; PEGA: pustulose exantemática generalizada aguda; IM: intramuscular; SDRIFE: *symmetrical drug-related intertriginous and flexural exanthema*.

Entre as contraindicações relativas para o teste, estão asma mal controlada e urticária ativa, e, dentre as absolutas, farmacodermias graves e anafilaxia quase fatal.^(1)^

Existem diferentes protocolos para a realização do TP, mas, frequentemente, é feito em duas ou três etapas (placebo, 10% da dose, seguida por 90% da dose após 30 a 60 minutos).^(44)^ A amoxicilina é o BL preferido para o teste, devido à presença do anel BL e da cadeia lateral R1.^(2)^ Na suspeita de alergia à amoxicilina-clavulanato, utilizar essa medicação no TP.^(2)^

Outra abordagem que tem sido avaliada recentemente é a realização do TP com BL, sem testes cutâneos prévios. Alguns estudos demonstraram a segurança dessa abordagem em crianças com história de exantema maculopapular à amoxicilina,^(45)^ e outros foram realizados também em adultos.^(46)^ Entretanto, um dos critérios de exclusão utilizados nesses trabalhos foi história de reação grave (por exemplo: anafilaxia), que contraindica a provocação direta.

Recentemente, nosso grupo publicou dois documentos descrevendo as indicações, as contraindicações e as técnicas recomendadas para a realização dos testes cutâneos e provocação com fármacos.^(43,47)^ Neles, podem ser encontradas mais informações a respeito desses testes. A tabela 3 resume os principais testes *in vivo* e *in vitro* para investigação de hipersensibilidade a BL.

**Tabela 3:** Testes *in vivo* e *in vitro* para investigação da hipersensibilidade a beta-lactâmicos

Classificação clínica da reação	Testes *in vivo*	Testes *in vitro*
Reações imediatas	Teste cutâneo de puntura (leitura imediata)	Triptase
	Teste cutâneo intradérmico (leitura imediata)	IgE específica
	Teste de provocação	Teste de ativação basofílica
Reações não imediatas	Teste de contato	Teste de transformação linfocítica
	Teste intradérmico (leitura tardia)	ELISPOT
	Teste de provocação	

ELISPOT: *enzyme-linked immunosorbent spot.*

## TRATAMENTO

A primeira conduta diante da suspeita de uma reação ao BL é suspender a exposição ao medicamento.^(3)^ A terapêutica deve ser realizada de acordo com a síndrome clínica apresentada pelo paciente. O único tratamento específico para hipersensibilidade a BL é a dessensibilização, que possui indicações bem precisas.

Em caso de urticária e/ou angioedema agudos, o anti-histamínico H1 é a medicação de primeira escolha, pois a histamina é o principal mediador envolvido.^(48)^ Deve-se dar preferência para os anti-H1 de segunda geração, considerando seu melhor perfil de segurança.

Na anafilaxia (reação de hipersensibilidade sistêmica imediata grave que pode levar à morte), o reconhecimento precoce e o tratamento rápido são fundamentais. A epinefrina é considerada a droga de primeira linha para o tratamento da anafilaxia, e sua prescrição precoce é essencial para reversão do quadro.^(49,50)^

A conduta diante de um quadro de anafilaxia é:^(49,50)^ suspender a exposição ao medicamento; avaliar o paciente (vias aéreas, respiração, circulação e estado mental); posicionar o paciente em decúbito dorsal e, se possível, elevar os membros inferiores; chamar ajuda de uma equipe de emergência; administrar epinefrina 1:1000 (1mg/mL) em doses de 0,01mg/kg (dose máxima de 0,3mg em crianças e 0,5mg em adultos) via intramuscular, preferencialmente no músculo vasto lateral da coxa; manter a via aérea pérvia, administrar oxigênio suplementar com máscara facial (fluxo 6 a 8L/minuto) e beta-agonistas inalatórios, se houver broncoespasmo; repor fluidos intravenosos (soro fisiológico 0,9%) e administrar drogas de segunda linha, como anti-histamínicos e corticosteroides.

### Dessensibilização

Quando um paciente alérgico a determinada medicação necessita dessa mesma droga para uso futuro e não há substituto custo-eficaz, uma alternativa é a dessensibilização. Trata-se de um procedimento que permite aos pacientes tolerar transitoriamente a medicação que desencadeou a reação original, com administração da dose completa para tratamento.^(5,51,52)^

A dessensibilização é uma ferramenta preciosa na gestão das RHM, notadamente as imediatas, incluindo anafilaxia, mas também em algumas não imediatas não graves. Pode ser utilizada no tratamento de qualquer reação de hipersensibilidade imediata, alérgica ou não alérgica. Nas RHM imediatas, é utilizada, em geral, a dessensibilização rápida à droga (DRD).^(51,53)^

A DRD induz, num curto período de tempo, à falta de resposta temporária a um fármaco particular que anteriormente tinha induzido a uma reação de hipersensibilidade, permitindo que o paciente seja exposto de forma segura ao fármaco culpado. Essa falta de resposta temporária pode ser obtida pela reintrodução gradual de pequenas doses do fármaco envolvido até a dose-alvo total, reduzindo notavelmente o risco de RHM graves e potencialmente letais.^(51)^ As evidências sugerem que as células efetoras de anafilaxia, mastócitos e basófilos tornam-se transitoriamente hiporresponsivas.^(54)^ A tolerância clínica foi descrita como ocorrendo dentro de algumas horas em pacientes submetidos à DRD – um tempo que não permite a indução de tolerância ao nível das células T. Ainda não está estabelecido se a DRD repetida em pacientes alérgicos a fármacos poderia induzir células T reguladoras após múltiplas dessensibilizações. Já os mecanismos que explicam a eficácia da dessensibilização nas reações tardias não são conhecidos. De maneira geral, a DRD consiste na administração consecutiva de pequenas doses do fármaco culpado até que a dose terapêutica total seja atingida.

As indicações de dessensibilização a drogas são:^(53)^ não há nenhuma droga alternativa; a droga envolvida é mais eficaz (maior qualidade de vida e/ou expectativa de vida) e/ou está associada a menos efeitos colaterais do que drogas alternativas e a droga culpada tem um mecanismo único de ação, como o ácido acetilsalicílico na doença respiratória exacerbada por essa substância.

O procedimento é indicado com precaução em pacientes de alto risco, e absolutamente contraindicado em reações tardias graves, com risco de vida, como síndromes de dermatite esfoliativa, SSJ, NET, DRESS, erupção fixa a droga, eritema multiforme, dermatite bolhosa, PEGA, reações imunocitotóxicas graves e vasculite. Isso significa que, depois de confirmar o diagnóstico de RHM, o alergista deve avaliar o risco do doente e a relação risco-benefício de uma dessensibilização. Quando a dessensibilização é indicada, deve-se obter um Termo de Consentimento Livre e Esclarecido.^(54)^

A eficácia da dessensibilização nas reações tardias não está tão bem documentada como nas reações imediatas, e os protocolos também são bastante variados, desde lentos até rápidos. A primeira DRD foi descrita com penicilina, durante a Segunda Guerra Mundial.^(55)^ Desde então, o procedimento foi utilizado para várias classes de drogas, tanto por via oral como injetável. Dentre os BL, a própria penicilina é o principal exemplo de antibiótico que pode ter uma indicação precisa e sem substituto clinicamente eficaz, como no caso da sífilis, em especial quando acomete uma gestante.^(53)^ Pacientes com alergia à penicilina IgE-mediada, incluindo anafilaxia, que necessitam de penicilina como terapia de primeira linha são candidatos à DRD.^(14)^

As vias de administração oral e parenteral podem ser utilizadas para DRD, apresentando eficácia semelhante, com taxa de sucesso em torno de 100%.^(14)^ Alguns estudos sugerem que a via oral para os pacientes com alergia à penicilina pode ser mais segura, fácil e menos dispendiosa, embora nem sempre seja a mais apropriada. Existem protocolos que combinam DRD oral e intravenosa para beta-lactâmicos,^(56)^ e o protocolo por via intravenosa em 12 etapas divididas em três bolsas (diluição 1:100, seguida de 1:10 e, finalmente, na diluição padrão) também já foi descrito como seguro e eficaz, mesmo em pacientes pneumopatas graves com volume expiratório forçado em 1 segundo com menos de 1L.^(57)^

É crucial saber que a dessensibilização tem efeito temporário que dura pelo menos duas meias-vidas do medicamento, após a qual a dessensibilização precisa ser repetida. A penicilina benzatina de ação prolongada está associada a eventos adversos aceitáveis, uma a três semanas após a dessensibilização à penicilina.^(56)^ Porém, quando a dessensibilização é empírica, na ausência de testes cutâneos positivos, não define se um paciente é verdadeiramente alérgico à droga. Assim, recomenda-se o acompanhamento de um teste formal de alergia à penicilina após a conclusão do tratamento com penicilina.^(14)^

## A IMPORTÂNCIA DA “DESROTULAGEM” DA ALERGIA À PENICILINA

O diagnóstico equivocado de alergia à penicilina pode afetar o sistema de saúde de duas formas: com o falso rótulo de alergia, com aumento irreal da incidência e impacto nas opções de tratamento, e o falso rótulo de não alérgico, que pode ter importantes consequências na prescrição errônea de medicações, especialmente nas reações graves.^(58)^

O paciente com rótulo de “alergia à penicilina” é mais propenso a receber antibióticos de amplo espectro, como fluoroquinolonas, vancomicina e clindamicina,^(9,59,60)^ e apresenta maior risco de infecções por *Clostridium difficile, Staphylococcus aureus* resistente à meticilina e *Enterococcus* resistente à vancomicina.^(9,59,60)^ Além da resistência antimicrobiana, estudos demonstraram que pacientes “alérgicos à penicilina” possuem maior risco de complicações pós-operatórias, tempo mais longo de internação hospitalar, custo mais elevado de tratamento e maior índice de falha terapêutica.^(7,61,62)^ A tabela 4 reúne as principais implicações para a saúde do indivíduo e a saúde pública do rótulo de “alergia a BL”.

**Tabela 4:** Implicações para a saúde pessoal e pública do rótulo de “alergia a beta-lactâmicos”

Implicações para a saúde do indivíduo	Implicações para o sistema de saúde pública
Menos opções de antibióticos eficazes	Resistência antimicrobiana
Mais efeitos tóxicos associados com o uso de antibióticos alternativos	Altas taxas de infecção por *Clostridium difficile*
Uso de antibióticos de amplo espectro	Uso de antibióticos mais caros
Mais infecções pós-cirúrgicas	Aumento do tempo de internação hospitalar

Fonte: traduzido e adaptado: Castells M, Khan DA, Phillips EJ. Penicillin Allergy. N Engl J Med. 2019;381(24):2338-51. Review.^(14)^

O diagnóstico exagerado de alergia a BL decorre de relatos superestimados, tanto por parte de médicos e/ou outros profissionais de saúde, quanto de pacientes ou seus responsáveis. Alguns sintomas (por exemplo: intolerância gastrintestinal e cefaleia) são falsamente considerados alérgicos.^(2)^ Além disso, uma reação cutânea (por exemplo: exantema maculopapular ou urticariforme) pode ser devida à interação entre o BL e o vírus, ou mesmo ter sido causada pela infecção viral.^(2)^ Outras considerações que podem influenciar no diagnóstico são a sensibilidade “baixa/subótima” dos testes cutâneos, e a história natural da alergia à penicilina.^(2)^ Ocorre um decréscimo natural dos anticorpos IgE contra a penicilina com o decorrer do tempo.^(2)^

A investigação precisa, por meio de testes adequados e acurados, é primordial para o manejo apropriado das reações de hipersensibilidade aos BL.^(57)^ Tradicionalmente, envolve tanto testes cutâneos quanto TP. Entretanto, o TP direto sem testes cutâneos prévios tem sido cada vez mais utilizado, em pacientes de baixo risco (sintomas cutâneos leves), objetivando simplificar e diminuir o custo dos procedimentos diagnósticos.^(45,63)^ Um estudo americano estimou o custo da investigação por meio de testes cutâneos e TP com penicilina em 220 dólares americanos. Em contrapartida, o TP direto teria um custo de 84 dólares americanos.^(63)^

Algumas estratégias conhecidas como “*pathways*” têm sido implantadas em hospitais ao redor do mundo para auxiliar na prescrição de antibióticos em pacientes com rótulo de “alergia aos BL”.^(1,64)^ Esses *pathways* são abordagens padronizadas que envolvem uma equipe multidisciplinar (gestores, médicos alergistas, infectologistas e clínicos gerais, enfermeiros e farmacêuticos) e buscam o uso racional de antibióticos.^(1,64)^ Os resultados obtidos geralmente incluem menor utilização de antibióticos não BL, com menor prevalência de infecção por germes resistentes; redução do tempo de internação hospitalar; menores taxas de mortalidade e redução significativa dos custos hospitalares.^(1,64)^

Outros aspectos fundamentais são a orientação e a identificação adequada do paciente submetido à investigação completa. Muitas vezes, mesmo após um TP negativo, o paciente mantém o rótulo de alérgico nos prontuários médicos.^(65)^ Em certas ocasiões, médicos não utilizam a medicação novamente, pois não consideram o fármaco “seguro”.^(65)^ Assim, a educação dos pacientes e seus responsáveis, além de médicos não especialistas, constitui um dos pilares da “desrotulagem” da alergia à penicilina.^(66)^

## PARTICULARES DA ALERGIA A BETA-LACTÂMICOS EM CRIANÇAS

O estudo das RHM em crianças apresenta grandes lacunas relacionadas à epidemiologia, aos aspectos clínicos e aos métodos diagnósticos. A maior parte das crianças com suspeita de RHM não é verdadeiramente alérgica a fármacos. Existem cinco diretrizes para RHM em adultos e apenas uma para a faixa pediátrica, e experiências em adultos são frequentemente extrapoladas para crianças.^(45,67)^

A causa mais comum de RHM na idade pediátrica são os antibióticos, principalmente os BL. Nessa faixa etária existem períodos em que infecções são mais frequentes (principalmente as infecções virais) e, dessa forma, podem mimetizar alergias a drogas.^(45)^

A avaliação precisa de RHM a antibióticos é parte essencial dos esforços do programa de administração de antibióticos, uma vez que a eliminação de rótulos de um paciente com suspeita de alergia a medicamento pode reduzir as exclusões desnecessárias. Existem inúmeras publicações recentes com realização de TP, utilizando BL sem realização de testes cutâneos em indivíduos, especialmente crianças, com eritema maculopapular leve ou urticária de aparecimento tardio, para orientar a decisão de reexposição.^(68,69)^ Finalmente, em artigo recente, a força-tarefa pediátrica da *European Academy of Allergy and Clinical Immunology* (EAACI), juntamente da *British Society for Allergy & Clinical Immunology* (BSACI), endossou a realização do TP nas manifestações citadas.^(1)^

No TP, um décimo da concentração total do BL deve ser administrado inicialmente e, se tolerado, uma dose completa deverá ser administrada por 1 a 7 dias após, dependendo do intervalo de tempo da reação índice (tempo entre a administração do BL e o início da reação referida). Num TP, uma reação não imediata pode ser excluída se, após a realização da dose terapêutica, tiver decorrido intervalo de tempo igual ao da reação de índice sem o aparecimento de sintomas. Os pacientes também são aconselhados a voltar, relatar se ocorreu alguma reação nesse intervalo de tempo e, de preferência, mostrar fotografias.^(1)^

O teste cutâneo de penicilina é seguro e eficaz na avaliação de crianças com histórico de alergia à penicilina. Quando o diagnóstico é de crianças com histórico de erupção cutânea benigna, mas sem relato de anafilaxia, os estudos realizados até o momento que examinaram as provocações diretas à penicilina foram realizados por especialistas ou em unidades de atendimento de urgência, e é desconhecida a segurança de tais provocações quando realizadas em clínicas não especializadas e em populações adultas.^(14)^

## CONCLUSÃO

Os beta-lactâmicos são a causa mais frequente de hipersensibilidade a fármacos mediada por mecanismo imunológico. O rótulo de “alergia a beta-lactâmico” constitui grande problema de saúde pública, pois leva ao uso de antibióticos alternativos, menos eficazes, mais tóxicos, mais caros e que podem contribuir para o aumento da resistência bacteriana. Desse modo, novas estratégias têm sido elaboradas, com o objetivo de aprimorar a investigação das reações de hipersensibilidade a beta-lactâmico. O uso do teste de provocação direto em pacientes de baixo risco tende a aumentar nos próximos anos, mas deve ser sempre supervisionado por alergistas treinados. Além disso, o desenvolvimento de programas educacionais, tanto para o público leigo quanto para profissionais de saúde não especialistas, com o objetivo de melhorar o reconhecimento, o diagnóstico e o tratamento de reações de hipersensibilidade a beta-lactâmico, é de suma importância.
